# Inequitable morbidity and injuries burden among informal sector workers in an urban area in Dhaka: a retrospective analysis of Médecins Sans Frontières occupational health clinics, Bangladesh, 2014–2023

**DOI:** 10.1186/s12889-025-26046-0

**Published:** 2025-12-31

**Authors:** Grazia Caleo, Sohana Sadique, Debbie Malden, Martins Dada, Jobin Joseph, Kalyan Velivela, Salim Mahmud Chowdhury, Christabel Mayienga, Tasmia Shenjuti, Gayathrie Sadacharamani, Rezwanur Rahman Masum, Mark Sherlock, Hamza Atim, Patrick Keating

**Affiliations:** 1 Médecins Sans Frontières, London, UK; 2 Médecins Sans Frontières, Dhaka, Bangladesh; 3 University of Saskatchewan, Saskatoon, Canada; 4 Médecins Sans Frontières, Amsterdam, The Netherlands; 5 Médecins Sans Frontières, Nairobi, Kenya; 6 Centre for Injury Prevention and Research, Dhaka, Bangladesh

**Keywords:** Occupational health, Injuries, Musculoskeletal, Workers’ rights, Bangladesh, Informal sector workers

## Abstract

**Introduction:**

In Bangladesh, 85% of the workforce is employed in the informal sector, characterised by unsafe working conditions, low wages, and a lack of social and labour protections. Evidence on the health and occupational injuries faced by informal sector workers is limited. This study assessed the health status of informal sector workers in Dhaka’s Kamrangirchar area, where Médecins Sans Frontières (MSF) has provided occupational health (OH) services and provides evidence to inform policy.

**Methods:**

We conducted a retrospective analysis using OH data from two MSF clinics, covering patients aged ≥ 18 years from February 2014 to December 2023. We performed a descriptive analysis, stratified by sex, using chi-squared tests to identify differences in health status by key characteristics, including patient demographics such as age and sex, factory type, work-related morbidity, injuries, nutritional status, and mental health. The analysis was limited to new consultations.

**Results:**

Between 2014 and 2023, 64,467 OH consultations occurred among adults aged ≥ 18 years, of which 23,874 were new consultations with sex data available (self-reported); 38.6% were women. Women were more likely to work in plastics (35.7% vs. 24.4%) and garment (28.8% vs. 18.9%) factories, whereas men were predominated in leather factories (17.5% vs. 7.8%). Machinery operation was reported by 92.8% of men and 91.5% of women. Work-related conditions accounted for 90.5% of all visits. The most common diagnoses for both sexes were musculoskeletal disorders (30.3%) and gastrointestinal conditions (22.7%). Injuries represented 4.3% of new consultations, with a higher proportion in men (6.0% vs. 1.8%), and 60% of injuries occurred in metal factories. Malnutrition affected 16.7% of men and 12.5% of women. Among 561 patients with mental health outcomes, mood disorders were more frequent in women (92% vs. 84%).

**Conclusions:**

This study highlights the significant work-related health burden faced by urban informal sector workers operating in hazardous environments, with gender-based differences. Urgent gender-sensitive workspace safety, social and mental health, and nutritional initiatives are needed. Ratifying the International Labour Organization Conventions C189 and C190, which aim to provide informal workers with the same protection as those in the formal sector, would be a crucial step toward safeguarding workers’ rights and well-being.

**Supplementary Information:**

The online version contains supplementary material available at 10.1186/s12889-025-26046-0.

## Introduction

Work is a recognized social determinant of health, yet millions of people worldwide experience illness or injury due to unsafe working conditions [1]. In 2019, the global burden of work-related diseases and injuries was estimated at 2.9 million, including over 300,000 deaths from occupational accidents [2, 3].

The International Labour Organization (ILO) and the World Health Organization (WHO) estimate that 26.44 million disability-adjusted life years (DALYs) are attributable to occupational injuries, with Asia and the Pacific region accounting for the second highest proportion of work-related deaths globally [3]. Informal employment dominates the labour market in this region, with approximately 1.3 billion people (68% of the total workforce) engaged in the informal sector [4].

In Bangladesh, this figure is even higher, with 85% of the workforce (around 51.7 million people) employed informally [4]. Informal workers often face hazardous conditions, low wages, and minimal social protections, yet they are closely linked to the formal economy, producing goods and services that support the broader population [5].

For instance, the informal economy in Bangladesh contributes 49–64% of the country’s total gross domestic product (GDP); however, despite the significant economic role played by the informal sector, it remains largely invisible and is characterised by unregulated small enterprise operating outside conventional labour framework, hazardous working conditions, low pay, and a lack of social security net programmes (e.g. occupation injury and sickness benefits or health insurance) or workplace rights [6, 7].

Evidence from the 2016–2017 Bangladesh Labour Force survey shows that occupational injuries affected 1.8% of textile and garment workers (most of whom were in the informal sector) rising to 12.3% among jute factory workers [8]. ILO conventions 189 and 190 seek to provide the same legal and social protections to informal sector workers as formal sector workers, including protection from violence and harassments. However, these have yet to be ratified in Bangladesh [9].

Dhaka, the country capital, represents an important work destination where annually around 47,000 people migrate looking for job opportunities, often in the informal sector where low skills are needed [10].

Since 2013, in this urban context, Médecins Sans Frontières (MSF) has provided free-of-charge occupational healthcare to informal sector factory workers in Kamrangirchar, a peri-urban area of Dhaka. In this area, a large, unofficially estimated workforce is engaged in multiple different informal manufacturing processes such us plastics recycling, garment-making, and metal fabrication [7, 11]. A previous qualitative study of health-seeking behaviour among Kamrangirchar dwellers, including informal sector workers, reported the hardship choice of prioritising work over health and wellbeing, due to long and intense labour, limited economy autonomy, constant fear of losing work, and income [12]. The study participants highlighted that these choices were made in the context of a fragmented care system with a wide range of service providers, including private clinics, non-governmental organisations, alternative practitioners and government hospitals, leaving patients often confused by contradictory recommendations/diagnoses [12].

Previous studies in Bangladesh and the wider region have shown that informal sector workers experience a range of health problems, including eye, respiratory, and musculoskeletal conditions, as well as work-related injuries (e.g., falls, cuts) and malnutrition [12–15]. However, evidence on the health status, work-related conditions, and occupational injuries among Bangladesh’s informal workforce remains limited, and there is a lack of detailed, nuanced data on key aspects such as morbidity patterns, injury burden, nutritional status, and mental health [13].

The present study addresses this gap by analysing occupational health data collected over multiple years from MSF clinics in Kamrangirchar, Dhaka, providing comprehensive insights into work characteristics, health outcomes, and gender-specific vulnerabilities among informal factory workers. These findings can inform labor, health equity, and social protection policies, as well as guide tailored models of care for this and similar underserved populations.

## Methods

### Study setting

Kamrangirchar, located on the Buriganga River in Bangladesh, is one of Dhaka’s largest peri-urban areas, with a population of 440,000 concentrated in an area of 4 km² [12, 14]. In Kamrangirchar, MSF has been running two occupational health clinics since 2013, providing medical care and psychological support to the workers from MSF-registered factories in the informal factory sector.

### MSF occupational health model of care

**Fig. 1:**
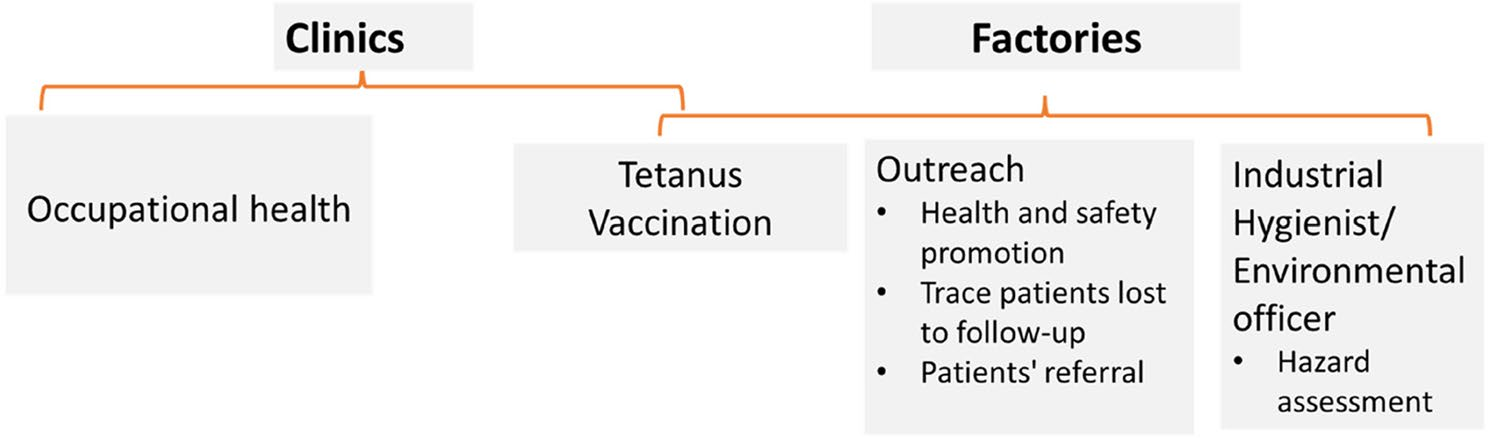
MSF Multidisciplinary model of care, Occupational Health, Dhaka, Bangladesh

To enhance access to care, MSF set up an agreement with factory owners/managers. The selection of factories targeted to receive occupational healthcare services was divided among four enterprises: tannery, garment, metal, and plastics. The willingness of the owner to use the MSF health facilities and to permit the workers to attend a 30-minute health consultation during the day were criteria for inclusion in the care programme. Once the agreement was established, factories and their workers were considered registered. MSF also provides care to workers in ‘unregistered’ factories and to the local population if they seek care from an MSF clinic. The occupational health model of care includes medical care, health promotion outreach activities, assessments of factory-work conditions, and safety training, along with tetanus vaccination performed inside the clinics and via mobile clinics inside the factories (Fig. 1). The MSF medical team received training in occupational health by qualified occupational health consultants, and an industrial hygiene specialist supported the team to assess and quantify hazards inside the factories and provided ad-hoc safety training [15]. Medical and hazardous work information gathered in the clinic helped to identify health promotion messages on how to protect against chemicals and prevent injury (Supplementary Fig. 1). Information collected inside the factories was used to support clinical management of patients (e.g. advice on ergonomic posture, proper handling of chemicals). The health promotion outreach team visited factories and informed factory owners/manager and workers about work occupational hazards and how to prevent exposure. The outreach team was also responsible for identifying patients who were lost to follow up and supporting patient’s referral.

### Study design

This study involves a retrospective analysis of anonymised occupational health consultation data from two MSF occupational health clinics in Kamrangirchar, Dhaka. Data for all occupational health patients (included workers from both registered and unregistered factories) aged 18 years or older who visited MSF Kamrangirchar clinics between February 28th, 2014, and December 31st 2023 were included in the analysis. Patients under 18 years were excluded from this analysis, as a separate analysis was conducted for this age group to specifically describe health and work-related outcomes associated with the worst forms of child labour [16].

### Data source and collection

Routine individual-level occupational health data collected in patient files during consultations with workers from both registered and unregistered factories were entered into a project-specific excel database. Diagnoses were classified using the ILO list of Occupational Diseases, and the World Health Organization’s (WHO) International Classification of Diseases (ICD-10) for occupational health [17, 18].

The following key variables were consistently collected over the study period and hence used in the current analysis: date of consultation, age, sex (self-reported), visit status (new/follow-up), residence inside a factory, type of factory they worked in, primary diagnosis and status of work-related morbidities, type and mechanism of injury, nutritional status, and mental health conditions.

New consultations were defined as patients who came for the first time for a consultation. An individual could appear more than once as a new consultation if the patient came on separate occasions with new conditions. Follow-up consultations were defined as patients who came for subsequent appointments (scheduled or walk-ins) to reassess and manage the patient’s ongoing occupational health condition.

Living inside the factory was defined as adults reporting the factory as their primary residence, defined as where they sleep. This variable is important as it might expose workers to additional environmental hazards (e.g., noise, chemicals), while also limiting mobility and social interactions. Moreover, workers who live on-site may be subject to extended working hours, increasing the risk of overexertion, fatigue, harassment and occupational accidents.

Work-related morbidity refers to any physical or psychological health conditions that are directly caused or exacerbated by the patient’s job or working conditions. This includes illnesses, chronic conditions, injuries, and psychological distress that arise from factors such as repetitive strain, exposure to hazardous materials, workplace accidents, or stressful work environments. During consultations, patients reported their symptoms, and occupational health clinicians assessed whether these conditions were attributable to work. In the dataset, a “yes” for work-related morbidity indicates that the individual both reported and was diagnosed with a work-related condition, while “no” indicates the absence of such conditions.

Nutritional status among workers older than 18 years was assessed using either mid upper arm circumference (MUAC) or body mass index (BMI) [19]. Workers were classified as malnourished if their MUAC was less than 210 mm or if their BMI for age score was more than two standard deviations below the average. Other measures of nutritional status such as anaemia or stunting were not part of routine data collection for occupational health patients.

### Data analysis

Descriptive analysis of the occupational health data from patients 18 years and older was performed.

The analysis of the occupational health was restricted to new consultations, as they provided an indication of the burden of occupational morbidities. All analyses were stratified by sex to identify potential gender differences in health outcomes. New consultations missing data on sex were excluded from the analyses by sex. Chi-squared tests were used to assess differences in proportions. The analysis was performed using R Studio software (R v 4. 3.2) [20].

## Results

### Demographics

During the study period, there were 64,467 occupational health consultations at the MSF clinics that involved adults aged 18 years or older, of whom 26,614 (41.3%) were female. The overall median age of patients was 32 years (IQR: 25–42). Among the 64,467 consultations, 23,885 were new consultations, 29,596 were follow-up consultations, and an additional 10,986 consultations had a missing status. Of the 23,885 new consultations, 11 were missing data on sex, so the final number of consultations included in the analysis by sex was 23,874. Among the patients attending a new consultation, there were more male patients (14,660; 61.4%) than female patients (9214; 38.6%), the median age was lower in men than in women, but the proportion of people reporting living inside the factory was higher in men than in women (Table 1 and 20% vs. 0.7%, respectively). Between 2014 and 2023, there was an increase in the proportion of female patients attending the clinics from 24% in 2014 to 51% in 2021, which decreased to 44% in 2023 (Supplementary Fig. 2).

**Table 1:** Demographic characteristics of patients from new occupational health consultations, overall and by sex

	Overall (*N*=23,874)*	Female (*n*=9214)*	Male (*n*=14,660)*	*p*-value†
Median age (IQR)	30 (23, 40)	30 (25, 40)	28 (22, 38)	<0.001
Residence				<0.001
Lives outside factory	18,740 (88%)	8191 (99%)	10,549 (80%)	
Lives inside factory	2656 (12%)	61 (0.7%)	2595 (20%)	
Unknown	2478	962	1516	

Data are *n* (%), unless otherwise stated. Data collected from patients aged 18 years or older at MSF clinics in Kamrangirchar, in 2014–2023

* IQR* interquartile range, *MSF* Médecins Sans Frontières

*Overall total excludes 11 patients missing data on sex

†Wilcoxon rank sum test; Pearson’s Chi-squared test

### Place of work

Over a quarter (28.8%) of patients who came for the first time reported working in plastics factories, followed by metal (22.8%) and garment (22.7%) factories (Table 2). The proportions of patients working in plastics and garment factories were higher in women than in men (plastics: 35.7% women vs. 24.4% men; garments: 28.8% women vs. 18.9% men), whereas the proportion of patients working in leather factories was higher in men (17.5%) than in women (7.8%) (Table 2).

**Table 2:** Type of factory among new occupational health patients, overall and by sex

	Overall (*N* = 23,874)	Female (*n* = 9214)	Male (*n* = 14,660)	*p*-value†
Type of factory				< 0.001
Plastics	6845 (28.8%)	3282 (35.7%)	3563 (24.4%)	
Metal	5432 (22.8%)	1990 (21.7%)	3442 (23.5%)	
Garment	5407 (22.7%)	2646 (28.8%)	2761 (18.9%)	
Leather	3277 (13.8%)	715 (7.8%)	2562 (17.5%)	
Tannery	1303 (5.5%)	97 (1.1%)	1206 (8.2%)	
Rubber	540 (2.3%)	155 (1.7%)	385 (2.6%)	
Embroidery	377 (1.6%)	155 (1.7%)	222 (1.5%)	
Other	422 (1.8%)	123 (1.3%)	299 (2.0%)	
Chemical	170 (0.7%)	17 (0.2%)	153 (1.0%)	
Battery	34 (0.1%)	8 (< 0.1%)	26 (0.2%)	
Unknown	67	26	41	

Data are *n* (%), unless otherwise stated. Data collected from patients aged 18 years or over at MSF clinics in Kamrangirchar, in 2014–2023

*MSF* Médecins Sans Frontières

†Pearson’s Chi-squared test

### Machinery operation

22,038 (92.3%) of patients reported operating machinery as part of their work, with little differences observed by factory type (Supplementary Table 1). Similar proportions of male and female patients reported operating machinery in their workplaces (91.5% for women and 92.8% for men) (Supplementary Table 2). Over time, there has been an increase in the proportion of women operating machinery, whereas high levels of machine operation among men were generally reported across the study period (Supplementary Fig. 3).

### Primary diagnoses

**Fig. 2:**
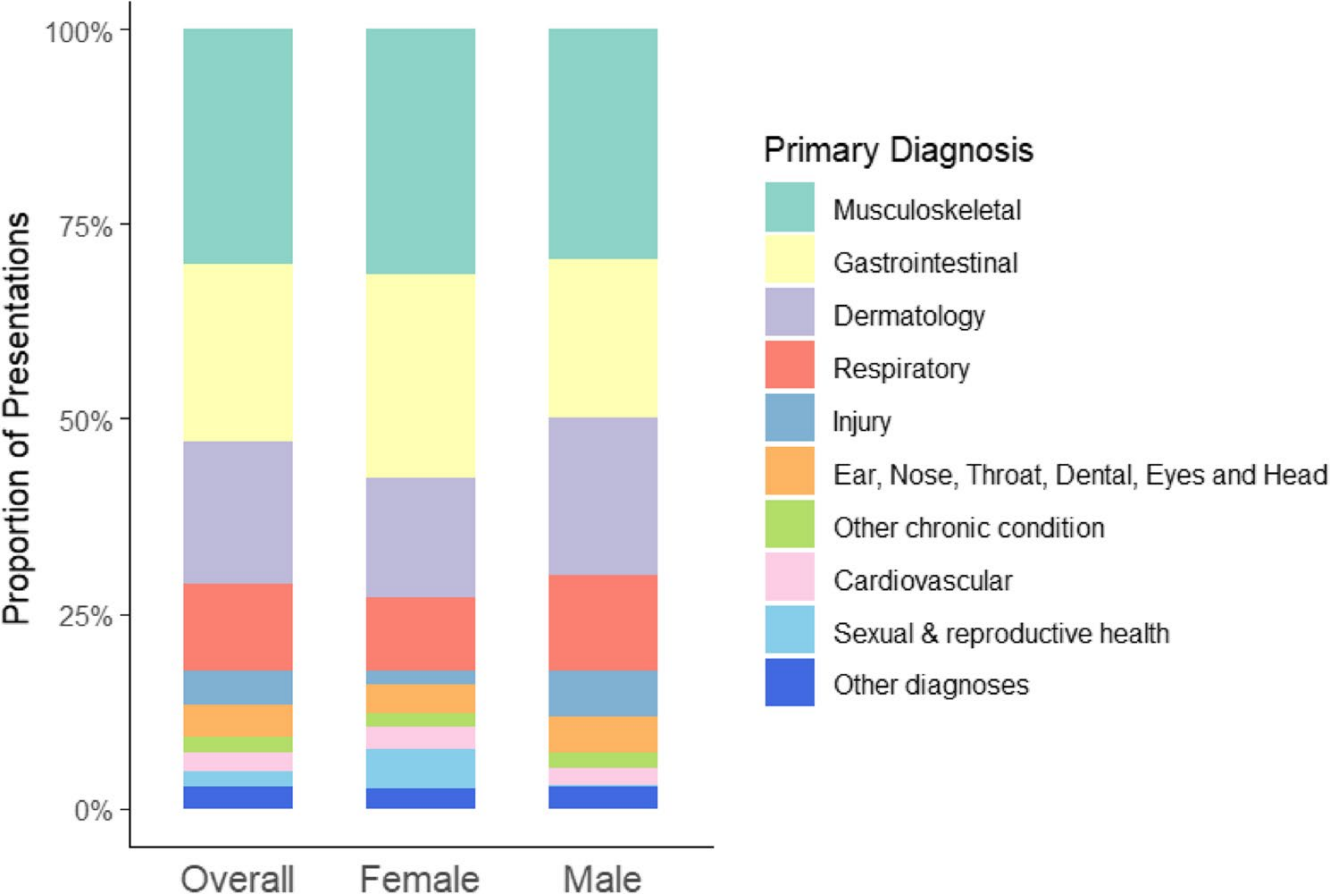
Primary diagnoses among new occupational health patients, overall and by sex*. * Unknown values are not presented and % are calculated excluding the unknown values totalling 842 patients

Musculoskeletal (30%) and gastrointestinal (23%) conditions together accounted for half of all diagnoses among adults, while 4.3% of new consultations were due to injuries (Fig. 2). These trends were broadly similar between female and male patients with a few exceptions including injuries, which represented 6.0% of male patient visits compared to 1.8% of female patient visits, and sexual and reproductive health (5% of female patient visits compared to 2% of male patient visits). Most conditions (90%) diagnosed during consultations were suspected as being work-related (Table 3), with little difference observed by most diagnosis types with some exceptions (non-communicable diseases, sexual and reproductive health, infectious diseases and neurological disorders) (Supplementary Table 3). 15% of all adults who sought care were malnourished, with a higher prevalence among male patients (17%) than female patients (15%) (Table 3). There was no difference in malnutrition status based on residence (living inside or outside the factory) (Supplementary Table 4). Compared with other age groups, the highest prevalence of malnutrition was observed among adolescents aged 18–19 years (31%).

**Table 3:** Suspected occupational condition and nutrition status among new occupational health patients, overall and by sex

	Overall (*N* = 23,874)	Female (*n* = 9,214)	Male (*n* = 14,660)	*p*-value†
Suspected occupational condition				< 0.001
Work-related	15,905 (90.5%)	6531 (89.2%)	9374 (91.4%)	
Non-work related	1677 (9.5%)	792 (10.8%)	885 (8.6%)	
Unknown	6292	1891	4401	
Nutrition status				< 0.001
Not malnourished (> 210 mm)	16,358 (84.9%)	6431 (87.5%)	9927 (83.2%)	
Malnourished (< 210 mm)	2913 (15.1%)	915 (12.5%)	1998 (16.7%)	
Unknown	4603	1868	2735	

Data are *n* (%), unless otherwise stated. Data collected from patients aged 18 years or over at MSF clinics in Kamrangirchar, in 2014–2023

*MSF* Médecins Sans Frontières

†Pearson’s Chi-squared test

### Mental health

Among the 561 mental health outcomes recorded, the primary mental health disorders diagnosed were mood-related (87%) with a higher proportion among females (92%) than among males (84%) (Table 4). Those aged 20–29 years accounted for most of the consultations (42%) (Supplementary Table 5). Key precipitating events included family disruptions, domestic violence, and socioeconomic issues, reported by over half of the patients (Table 4). Domestic discord and family violence was more prevalent among females (30%) than among males (14%). The proportion of male patients who received mental care having experienced abuse and sexual trauma was 2% compared to 1% among female patients.

**Table 4:** Characteristics of mental health outcomes among new occupational health patients, overall and by sex

	Overall (*N* = 561)	Female (*n* = 227)	Male (*n* = 334)	*p*-value†
Type of mental health disorder				0.002
Mood-related problems	489 (87%)	209 (92%)	280 (84%)	
Behaviour-related symptoms	27 (5%)	4 (2%)	23 (7%)	
Family-related problem	13 (2%)	2 (1%)	11 (3%)	
Physical complaints	12 (2%)	7 (3%)	5 (1%)	
Neuropsychiatric-related symptoms	13 (2%)	2 (1%)	11 (3%)	
Other	5 (1%)	3 (1%)	2 (1%)	
Social functioning	2 (0%)	0	2 (1%)	
Precipitating event				
Domestic discord & family violence	113 (21%)	67 (30%)	46 (14%)	
Socioeconomic functioning	96 (18%)	37 (17%)	59 (18%)	
Disruption of family & relationships	100 (18%)	56 (25%)	44 (13%)	
Medical illness-related	49 (9%)	10 (5%)	39 (12%)	
Neuropsychiatric-related	34 (6%)	4 (2%)	30 (9%)	
Events related to abuse during detention	13 (2%)	6 (3%)	7 (2%)	
Events related to natural disasters	11 (2%)	5 (2%)	6 (2%)	
Events related to violence	7 (1%)	2 (1%)	5 (2%)	
Deprivation, discrimination, and displacement	6 (1%)	2 (1%)	4 (1%)	
Sexual trauma or abuse	8 (1%)	3 (1%)	5 (2%)	
Others	111 (20%)	28 (13%)	83 (25%)	
Unknown	13	7	6	

Data are *n* (%), unless otherwise stated. Data collected from patients aged 18 years or over at MSF clinics in Kamrangirchar, in 2014–2023. Only a single mental health disorder and precipitating event was recorded at each consultation

*MSF* Médecins Sans Frontières, †Pearson’s Chi-squared test

### Injuries

Among the 1816 injuries reported across the study period, 537 were among new consultations. Most of these injuries reported in new consultations occurred in metal factories (60%) and among men (470 of 537 [88%]) (Table 5). Cut and lacerations accounted for almost half of all injuries (47%) and being struck was the primary mechanism of the injury (66%).

**Table 5:** Place and characteristics of injuries among new occupational health patients, overall and by sex

	Overall (*N* = 537)	Female (*n* = 67)	Male (*n* = 470)	*p*-value^†^
Type of factory				0.012
Metal	324 (60%)	30 (45%)	294 (63%)	
Plastics	110 (20%)	19 (28%)	91 (19%)	
Garment	38 (7.1%)	12 (18%)	26 (5.5%)	
Other	25 (4.7%)	3 (4.5%)	22 (4.7%)	
Leather	25 (4.7%)	2 (3.0%)	23 (4.9%)	
Embroidery	6 (1.1%)	1 (1.5%)	5 (1.1%)	
Rubber	5 (0.9%)	0 (0%)	5 (1.1%)	
Tannery	4 (0.7%)	0 (0%)	4 (0.9%)	
Chemical	0 (0%)	0 (0%)	0 (0%)	
Type of injury				0.3
Cut/laceration	225 (47%)	26 (43%)	199 (47%)	
Other	124 (26%)	21 (35%)	103 (24%)	
Crushing injury	51 (11%)	7 (12%)	44 (10%)	
Burn	42 (8.7%)	3 (5.0%)	39 (9.3%)	
Abrasion	24 (5.0%)	1 (1.7%)	23 (5.5%)	
Amputation	7 (1.5%)	0 (0%)	7 (1.7%)	
Broken bone	4 (0.8%)	1 (1.7%)	3 (0.7%)	
Bruise	4 (0.8%)	1 (1.7%)	3 (0.7%)	
Concussion	0 (0%)	0 (0%)	0 (0%)	
Unknown	56	7	49	
Mechanism of injury				> 0.9
Struck*	351 (66%)	44 (68%)	307 (66%)	
Other	94 (18%)	10 (15%)	84 (18%)	
Fall	45 (8.5%)	5 (7.7%)	40 (8.6%)	
Burn	19 (3.6%)	3 (4.6%)	16 (3.4%)	
Caught in between	20 (3.8%)	3 (4.6%)	17 (3.7%)	
Unknown	8	2	6	

Data are *n* (%), unless otherwise stated. Data collected from patients aged 18 years or over at MSF clinics in Kamrangirchar, in 2014–2023

*MSF* Médecins Sans Frontières. †Fisher’s exact test

* Refers to being struck by the machinery or a tool

## Discussion

Our study provides critical visibility to an underserved workforce in the informal sector, who bear significant work-related morbidities and injuries in a peri-urban area of Dhaka, Bangladesh. To our knowledge, this is the largest study to report on morbidities and occupational injuries among urban informal workers seeking medical care in Bangladesh, with a particular focus on sex differences. The increasing proportion of female patients attending clinics—rising from 24% in 2014 to 51% in 2021—likely reflects a shift in workforce demographics and highlights a growing need for gender-specific occupational health services.

The data reflects that Bangladesh still faces substantial challenges in achieving universal health coverage, particularly for its informal sector workers, who make up 85% of the labour force, and who contribute significantly to the national GDP [21]. These workers, working often in hazardous conditions with limited access to formal healthcare, bear high out-of-pocket healthcare costs. For instance, a study on 557 informal workers in Bangladesh, including rickshaw pullers, shopkeepers, and restaurant workers, revealed that 57% had fallen ill in the past 6 months, incurring health expenses that took up 8.9% of their annual income [22]. Additionally, income loss due to missed work took 28.5%, resulting in a combined financial burden of 37.4% of annual income [22]. This high financial burden increasesvulnerability and deepens social disparities, as informal workers lack social and occupational protections like sick leave, health insurance, and workspace safety [9]. Additionally, the informal sector does not contribute to taxes, which limits public resources for essential services [5]. Expanding protections, including ratification of ILO Conventions 189 and 190, would not only improve the wellbeing of informal workers, but also encourage formalisation, leading to tax contributions that could benefit the broader population by supporting public health, infrastructure, and social services [9].

Among workers who sought care at MSF clinics, musculoskeletal (30%) and gastrointestinal diseases (23%) were the most commonly documented conditions. This finding is consistent with other research in Bangladesh and other countries identifying these as common morbidities among informal industrial workers, which are likely due to poor working environments including lack of water and sanitation, or inappropriate personal protective equipment (PPE), long hours and repetitive motions, heavy lifting, exposure to dust, silica, and fumes, which are often under-regulated in workplaces [23–31]. These morbidities and others identified may impact the future employment of workers if they are out of work for extended periods to recover and/or unable to continue working as a result of these conditions [31]. In addition, musculoskeletal disorders were among the leading causes of years lived with disability in Bangladesh according to the 2019 Global Burden of Disease Study highlighting their public health significance [32].

In particular, workers in metal factories had a high frequency of injuries consistent with other quantitative and qualitative assessments of occupational hazards in Bangladesh and India [25, 33, 34]. Irrespective of factory type, most workers used some form of machinery. Data on the need, availability, and acceptability for PPE were not available in this study. However, other studies in Bangladesh have shown most workers understand the need for PPE and used some PPE, but PPE had more limited usefulness due to its inconsistent availability or ill-fitting/inappropriate nature [25, 28]. Similarly, a study on garment workers in India highlighted low use of PPE due to lack of availability and the impact on worker’s productivity [35].

While representing less than 5% of the primary diagnoses in this analysis, occupational injuries can have serious short and long-term consequences for workers. In the short term, injured factory workers may face high medical costs to treat their injuries and lose their salary for the days they don’t work, ultimately exposing them to high financial risk [21, 22, 36]. In the longer term, they may be unable to continue working (e.g. due to loss of fingers/limbs), which may push them further into extreme poverty [31, 33].

However, while acute injuries were more common in metal factories, the high prevalence of musculoskeletal complaints reflects the cumulative “wear-and-tear” associated with physically demanding tasks, repetitive movements, prolonged standing, and suboptimal ergonomic conditions. The repetitive nature of machinery operation across all factory types suggests that musculoskeletal strain and chronic morbidity are widespread, not confined to any single type of factory. Over time, this cumulative physical strain and poor working conditions can lead to chronic disability, reduced earning capacity, and extended periods out of work, highlighting the importance of preventive interventions that go beyond acute injury management.

The proportion of malnourished factory workers in this study was similar to findings from the Demographics and Health Survey (DHS) conducted in Bangladesh in 2022, with 20% of men aged 20–49 being underweight compared to 17% in this study and 9% of women aged 20–49 being underweight compared to 12% in this study [37]. A large proportion of factory workers in this study were diagnosed as being malnourished (15%). This important malnutrition issue was also identified in other studies in garment workers in Bangladesh and India [38, 39]. Khatun et al. used anaemia as a proxy for malnutrition and found substantially higher levels of anaemia among female workers compared with male workers (77% vs. 11%) in a garment factory [38]. The sex-specific differences in malnutrition observed between their study and ours may be explained by the use of different measures of malnutrition [38]. Importantly, our study identifies age-related differences in malnutrition: adolescents aged 18–19 years had the highest prevalence (31%) compared with 18% and 11% among those aged 20–29 years 30–49 years, respectively This finding, alongside the observed sex differences, highlights that both age and gender are key determinants of nutritional vulnerability in the informal workforce. These results emphasise the urgent need for nutrition interventions tailored to both adolescents and adult workers, alongside improved working conditions, to enhance the health outcomes of factory workers [40].

In this study, a large number of patients were diagnosed with mental health issues (*n* = 561), including mood and behaviour related problems and most of these patients were aged 20–29 years. Several studies have described workplace psychological hazards in Bangladesh factories and particularly among ready-made garment factories, including job insecurity, long hours, unsafe work environments, lack of security at the workplace, sexual harassment, and low income [41–43]. These occupational psychological stressors as well as the financial stress can lead to mental health problems such as depression and anxiety. Workers’ occupational psychological hazards may be further compounded by the stressors in their home environments. These broader findings highlight the need for comprehensive mental health support as part of occupational health interventions.

Our findings highlight significant gender differences. The proportion of patients who reported living inside the factories was higher in men than in women, suggesting that men may face unique health and safety risks associated with living directly in the work environment, such as prolonged exposure to hazardous environment, aluminium dust, and a lack of separation between work and personal life [44, 45]. Although similar proportions of men and women reported working with machines, men were more frequently involved in high-risk activities, such as heavy machine operation, which may explain the higher incidence of work-related injuries. We also observed a higher proportion of malnourished patients in men, particularly among those aged 18–19 years. In contrast, women exhibited a higher prevalence of mood-related problems. In summary, these gender-related differences emphasise the need for tailored occupational health policies that consider gender-specific risks, including tetanus immunisation, targeted safety training, injury prevention measures, nutrition and social and mental health interventions [46, 47].

This study has several limitations and strengths. The programmatic nature of the occupational health dataset was a key limitation. As is common with many programmatic datasets, there were a number of data quality issues that may have impacted the validity of the findings, including a large amount of missing data on consultation status (17%), and a lack of detail on the machinery used (e.g. no differentiation from use of sewing machines to more dangerous heavy machinery) by factory workers and associated availability of PPE. In addition, malnutrition in adults was not measured using standardised approaches such as WHO’s recommended fixed BMI thresholds or using the Malnutrition Universal Screening Tool, which could have led to some misclassification of patients [19, 48]. Moreover, we could not define the true population burden since we did not know the total number of occupational health conditions in the population. In addition, the analysis presents findings from factory workers who were able to access MSF health facilities, and they may not fully represent the occupational health needs of the broader population. For example, the lower representation of women in the dataset could be more of an access issue rather than a reflection on women’s occupational health needs in this population. Another limitation is the lack of data on the severity of injuries, which limits our understanding of immediate and long-term disability consequences. In addition, we do not have information on the number of follow-up consultations related to the morbidity diagnosed in the first visit due to a lack of analysis of follow-up consultation data. Contextual aspects of the workers are missing, making it difficult to understand the workers lived experience and faced challenges (e.g. information on working hours, salary and education status of the workers). Combined, these limitations make it difficult to have a comprehensive understanding of the occupational health needs and longer-term health consequences of this population.

This study does, however, have several strengths. The data presented have been collected by trained occupational health clinicians over a 10-year period providing an in-depth overview of the occupational health needs of the adult population. The study provides critical visibility to an underserved and often neglected population, offering insights directly relevant for occupational health interventions and policy.

The gender lens approach of the analyses identified key differences in occupational health needs between men and women, which can help inform gender-sensitive interventions. The findings on morbidities are in line with previously published work, while the inclusion of mental health data emphasises this important component of occupational health interventions. By documenting both acute injuries and chronic musculoskeletal strain, the study highlights the broader spectrum of occupational health risks, providing a comprehensive and policy-relevant overview that can guide prevention, care, and social protection strategies for this population.

Finally, data interpretation and recommendations have been informed by the occupational and outreach teams, who have worked on the project over the last 10 years, thus ensuring that conclusions are grounded in the real-world context of this workforce.

### Recommendations

This study provides critical insights into the occupational health challenges faced by urban informal workers in Bangladesh. We provide recommendations presented in Table 6 that could support tailored policy initiatives for this and similar workforce populations.

**Table 6:** Recommendations for improving occupational health in informal workplaces

Domains	Macro-Level (Structural & Policy Reforms)	Meso-Level (Workplace & Community Interventions)	Proximal-Level (Direct Impact on Workers)
Policy & Structural Reforms	• Ratify International Labour Organization (ILO) Conventions C189 and C190 to extend legal protections to informal workers. • Reduce costs & complexities of factory registration to support formalisation. • Establish Occupational Health Departments in secondary government healthcare facilities. • Appoint an Occupational Health and Safety (OSH) officer to coordinate factory assessments and compliance.	• Strengthen existing occupational safety policies and promote awareness of the benefits of formalisation. • Ensure regular factory non-punitive inspections for safety compliance.	• Inform workers about their labour rights, access to healthcare, and legal protections.
Unionization, Policy Advocacy, and Women's Participation in the Informal Sector	• **Promote Labour Unions in Informal Sectors:** Advocate for the establishment and recognition of labour unions in informal sectors, particularly for women workers. • **Legal Protections for Women in the Informal Sector:** Strengthen labour laws to include specific provisions for women in informal labour markets. This can involve the implementation of anti-discrimination laws, equal pay for equal work, and protections against sexual harassment and violence in workplaces. • **Support Gender-Responsive Unionisation Policies:** Support the creation of gender-sensitive labour union policies that address the specific needs of women in informal sectors.	• **Encourage Women’s active Participation in Union Leadership:** Organise training programmes and awareness campaigns to help women understand their rights and the benefits of being part of labour unions. • **Strengthen Collective Bargaining for Women Workers:** Ensure that labour unions advocate specifically for women’s needs during collective bargaining processes. This includes addressing issues such as maternity leave, flexible working hours, equal pay, and protection from Sexual and gender-based violence (SGBV) at the workplace. • **Establish Gender-Sensitive Support Mechanisms within Unions:** Labour unions can establish support systems, such as women-only spaces or support groups, to address the specific challenges faced by women in the informal sector.	• **Provide Women Workers with Access to Union Resources:** Ensure that women working in informal sectors are informed about the benefits of joining labour unions, providing them with direct access to union resources and support services, including legal assistance and health coverage. • **Support Women’s Health and Safety at Work:** Labour unions should offer direct health and safety training to female workers, especially in informal sectors. Specific attention should be given to reproductive health needs and the prevention of workplace violence. • **Create Awareness of Gender Equity in Union Roles:** Raise awareness about the importance of women’s participation in unions. This could include targeted campaigns or workshops within informal workplaces to foster the value of female representation within labour unions.
Workplace Safety & Occupational Health	• Develop national strategies to improve gender-sensitive occupational health policies in informal industries. • Promote industry-wide compliance with safety regulations.	• Create multi-disciplinary safety committees involving workers, factory owners, medical teams, and regulators. • Establish mobile health units or low-cost clinics near informal workplaces for occupational health services. • Strengthen factory-based health and safety monitoring. · Ensure tetanus is included in the package of prevention and care.	• Ensure gender-sensitive health interventions (e.g., maternal health for women, injury prevention for men in high-risk jobs). · Design hazard assessment and preventive measures specifically for young and adolescent workers. • Provide workers with PPE, first aid training, and safety awareness sessions.
	Include musculoskeletal health and ergonomic standards in national occupational health policies for informal sector workplaces, with attention to age- and sex-specific needs.	Promote regular ergonomic assessments in factories to identify risk factors for musculoskeletal disorders and implement corrective measures (e.g., workstation redesign, task rotation, rest breaks). Integrate training on safe lifting, posture, and micro-breaks for workers. Track Musculoskeletal complaints systematically to guide preventive strategies.	Implement targeted interventions to prevent and manage musculoskeletal strain, including access to physiotherapy, rehabilitation, and education on safe work practices.
Healthcare Access & Support Services	• Institutionalise occupational health as part of public health and social welfare strategies. • Support factory-linked health programmes through public-private partnerships.	• Set up specialised rehabilitation centres for workers with severe injuries or chronic illnesses. • Improve referral pathways for mental health and workplace injury treatment.	• Integrate mental health support as part of occupational health programmes. • Provide accessible healthcare services for workers facing chemical exposure, ergonomic injuries, and respiratory issues.
Nutrition & Worker Well-being	Develop national nutrition programs tailored to labour-intensive industries.	Develop national nutrition program including age- and adolescent-specific interventions	Actively involve young and adolescent informal workers in nutritional and wellbeing initiatives
Sexual & Gender-Based Violence (SGBV)	• Develop national policies ensuring workplace protections against SGBV in the informal sector.	• Integrate SGBV services into broader labour rights frameworks. • Establish contact points and referral systems for SGBV survivors to access One-Stop Crisis Centres. • Train factory managers, workers, and health personnel on SGBV prevention and response protocols. • Provide referral pathways and health awareness programmes for both male and female factory workers to enhance access to SGBV services.	• Provide contact points, including community based/grassroot organisations, with information and referral systems for those who are survivors of SGBV to One Stop Crisis Centres. • Posters and other adapted informational materials placed in factories and communities could enhance access to health and service provisions.
Research, Data, & Advocacy	• Conduct human and labour rights-based research to inform policy. • Document and disseminate best practices for informal sector health interventions. • Advocate for stronger labour protections and health policies.	• Use qualitative research to capture informal workers' lived experiences. • Strengthen workplace-based surveillance and reporting mechanisms for occupational diseases.	• Encourage worker participation in research initiatives to improve health and safety outcomes. • Encourage collaboration with community-based /grassroot organisations focused on worker’s rights to ensure greater impact and enhanced advocacy of research findings.

## Conclusions

This study highlights the significant work-related health burden faced by urban informal sector workers operating in hazardous environments, with important gender differences. Urgent gender-sensitive workspace safety, social and mental health, and nutritional initiatives are needed. Additionally, ratifying ILO Conventions C189, and C190, which aim to provide informal workers with the same protection as those in the formal sector, would be a crucial step toward safeguarding workers’ rights and well-being. This will ultimately benefit the overall population by fostering economic resilience, health and social equity, and positive public health outcomes.

## Supplementary Information

Additional file 1: Includes three figures and five supporting tables: (1) Example of a Health and safety promotion leaflet developed by MSF, Dhaka; (2) Proportion of new occupational health patients aged 18 years or older by sex (3) Type of factory among new occupational health patients by machine operation status; (4) Task in factory among new occupational health patients by sex; (5) Proportion of new occupational health patients by sex and task; (6) Primary diagnosis among new occupational health patients by work-related status of the diagnosis; (7) Residence and age group among new occupational health patients by nutrition status; (8) Distribution of new mental health patients by age group.

Additional file 2: Data dictionary for the variables included in this analysis of occupational health data from MSF clinics, Kamrangirchar, 2014–2023.

## Data Availability

The datasets supporting the conclusions of this article are available on request in accordance with MSF’s data sharing policy. Requests for access to data should be made to [oca.research@london.msf.org] (mailto: oca.research@london.msf.org).

## Abbreviations

BMI: Body Mass Index
ILO: International Labour Organization
MSF: Médecins Sans Frontières
MUAC: Mid-Upper Arm Circumference
OH: Occupational Health
PPE: Personal Protective Equipment
WHO: World Health Organization
