# Supplementary material for: Inequitable morbidity and injuries burden among informal sector workers in an urban area in Dhaka: a retrospective analysis of Médecins Sans Frontières occupational health clinics, Bangladesh, 2014–2023
Source: BMC Public Health. 2025 Dec 31;26:418. doi: 10.1186/s12889-025-26046-0 (PMC12865996; doi:10.1186/s12889-025-26046-0)
Supplement: Supplementary file 1 — Additional file 1: Includes three figures and five supporting tables: (1) Example of a Health and safety promotion leaflet developed by MSF, Dhaka; (2) Proportion of new occupational health patients aged 18 years or older by sex (3) Type of factory among new occupational health patients by machine operation status; (4) Task in factory among new occupational health patients by sex; (5) Proportion of new occupational health patients by sex and task; (6) Primary diagnosis among new occupational health patients by work-related status of the diagnosis; (7) Residence and age group among new occupational health patients by nutrition status; (8) Distribution of new mental health patients by age group. [file 12889_2025_26046_MOESM1_ESM.docx]

**Supplementary Figure 1: Example of a health and safety promotion leaflet developed by MSF, Dhaka**


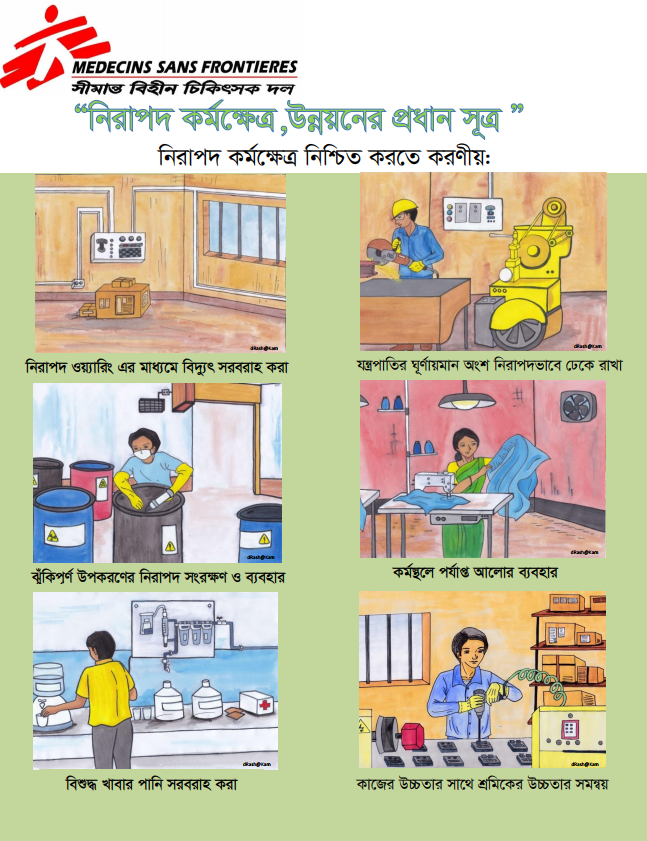


MSF=Médecins Sans Frontières.

**Supplementary Figure 2:** **Proportion of new occupational health patients aged 18 years or older by sex**


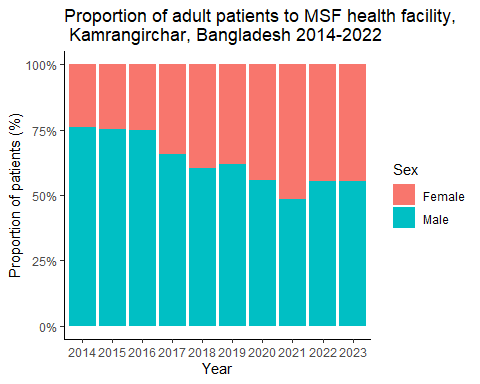


Data were collected from MSF clinics, Dhaka, 2014–2023. MSF=Médecins Sans Frontières.

**Supplementary Table 1: Type of factory among new occupational health patients by machine operation status**

| **Characteristic** | **Overall**  **(N = 23,855)** | **Operates machine**  (N = 22,0461) | **Does not operate machinery**  (N = 1,8391) |
| --- | --- | --- | --- |
| **Type of factory** |  |  |  |
| Plastics | 6,845 (28.7%) | 6,178 (90%) | 667 (9.7%) |
| Metal | 5,432 (22.8%) | 5,134 (95%) | 298 (5.5%) |
| Garment | 5,407 (22.7%) | 5,175 (96%) | 232 (4.3%) |
| Leather | 3,279 (13.7%) | 2,975 (91%) | 304 (9.3%) |
| Tannery | 1,312 (5.5%) | 1,073 (82%) | 239 (18%) |
| Rubber | 540 (2.3%) | 497 (92%) | 43 (8.0%) |
| Embroidery | 377 (1.6%) | 372 (99%) | 5 (1.3%) |
| Other | 422 (1.8%) | 406 (96%) | 16 (3.8%) |
| Chemical | 170 | 158 (93%) | 12 (7.1%) |
| Battery | 34 | 34 (100%) | 0 (0%) |
| Unknown | 67 | 44 | 23 |

Data are n (%). Data collected from patients aged 18 years or over at MSF clinics in Kamrangirchar, in 2014–2023. MSF=Médecins Sans Frontières.

**Supplementary Table 2:** **Task in factory among new occupational health patients by sex**

| **Characteristic** | **Overall**  **(N=23,874)** | **Female**  (n=9214) | **Male**  (n=14,660) |
| --- | --- | --- | --- |
| **Task in factory** |  |  |  |
| Operates machine | 22,038 (92.3%) | 8429 (91.5%) | 13,609 (92.8%) |
| Does not operate machinery | 1836 (7.7%) | 785 (8.5%) | 1051 (7.2%) |

Data are n (%). Data collected from patients aged 18 years or over at MSF clinics in Kamrangirchar, in 2014–2023.

**Supplementary Figure 3:** **Proportion of new occupational health patients by sex and task**


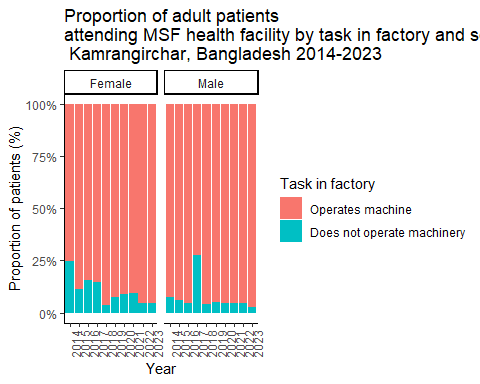


Data collected from patients aged 18 years or over at MSF clinics in Kamrangirchar, in 2014–2023. MSF=Médecins Sans Frontières.

**Supplementary Table 3:** **Primary diagnosis among new occupational health patients by work-related status of the diagnosis**

| **Characteristic** | **Overall (N=17,593)** | **Work-related**  (n=15,911) | **Non-work related**  (n=1682) |
| --- | --- | --- | --- |
| **Primary diagnosis** |  |  |  |
| Musculoskeletal | 5773 (32.8%) | 5502 (95%) | 271 (4.7%) |
| Gastro-intestinal | 3740 (21.3%) | 3382 (90%) | 358 (9.6%) |
| Dermatology | 3208 (18.2%) | 2979 (93%) | 229 (7.1%) |
| Respiratory | 1824 (10.4%) | 1604 (88%) | 220 (12%) |
| Injury | 785 (4.5%) | 752 (96%) | 33 (4.2%) |
| ENTDEH (ear, nose, throat, dental, eyes and head) | 784 (4.5%) | 707 (90%) | 77 (9.8%) |
| Cardiovascular | 358 (2.0%) | 331 (92%) | 27 (7.5%) |
| SRH (sexual & reproductive health) | 206 (1.2%) | 52 (25%) | 154 (75%) |
| Other chronic condition | 294 (1.7%) | 263 (89%) | 31 (11%) |
| Others | 224 (1.3%) | 101 (45%) | 123 (55%) |
| Infectious disease | 55 (0.3%) | 35 (64%) | 20 (36%) |
| Non-communicable disease | 66 (0.4%) | 30 (45%) | 36 (55%) |
| Urinary | 37 (0.2%) | 26 (70%) | 11 (30%) |
| Haematology | 16 (0.1%) | 10 (63%) | 6 (38%) |
| Neurological disorder | 29 (0.2%) | 15 (52%) | 14 (48%) |
| Mental health | 7 (<0.1%) | 5 (71%) | 2 (29%) |
| Unknown | 187 | 117 | 70 |

Data are n (%). Data collected from patients aged 18 years or over at MSF clinics in Kamrangirchar, in 2014–2023. Overall total excludes 6292 patients for which work-related information was not available

**Supplementary Table 4:** **Residence and age group among new occupational health patients by nutrition status**

| **Characteristic** | **Overall**  **(N=19,282)** | **Not malnourished**  (n=16,367) | **Malnourished**  (n=2915) | **p-value†** |
| --- | --- | --- | --- | --- |
| **Residence** |  |  |  | 0.054 |
| Lives outside factory | 14,989 (78%) | 12,748 (85%) | 2241 (15%) |  |
| Lives inside factory | 2070 (11%) | 1727 (83%) | 343 (17%) |  |
| Unknown | 2223 | 1892 | 331 |  |
| **Age group (years)** |  |  |  | <0.001 |
| 18–19 | 1943 (10%) | 1347 (69%) | 596 (31%) |  |
| 20–29 | 6853 (36%) | 5628 (82%) | 1225 (18%) |  |
| 30–39 | 5404 (28%) | 4886 (90%) | 518 (10%) |  |
| 40–49 | 2828 (15%) | 2545 (90%) | 283 (10%) |  |
| 50–59 | 1467 (8%) | 1260 (86%) | 207 (14%) |  |
| 60+ | 787 (4%) | 701 (89%) | 86 (11%) |  |

Data are n (%), unless otherwise stated. Data collected from patients aged 18 years or over at MSF clinics in Kamrangirchar, in 2014–2023. Overall total excludes 4603 patients for which nutrition status was not available. MSF=Médecins Sans Frontières. †Pearson’s Chi-squared test.

**Supplementary Table 5:** **Distribution of new mental health patients by age group**

|  | N=561 |
| --- | --- |
| Age group (years) |  |
| 18–19 | 72 (13%) |
| 20–29 | 238 (42%) |
| 30–39 | 142 (25%) |
| 40–49 | 85 (15%) |
| 50–59 | 18 (3.2%) |
| 60+ | 6 (1.1%) |

Data are n (%). Data collected from patients aged 18 years or over at MSF clinics in Kamrangirchar, in 2014–2023.
