# Supplementary material for: Inequitable morbidity and injuries burden among informal sector workers in an urban area in Dhaka: a retrospective analysis of Médecins Sans Frontières occupational health clinics, Bangladesh, 2014–2023
Source: BMC Public Health. 2025 Dec 31;26:418. doi: 10.1186/s12889-025-26046-0 (PMC12865996; doi:10.1186/s12889-025-26046-0)
Supplement: Supplementary file 2 — Additional file 2: Data dictionary for the variables included in this analysis of occupational health data from MSF clinics, Kamrangirchar, 2014–2023. [file 12889_2025_26046_MOESM2_ESM.docx]

Data dictionary for occupational health data for patients 18 years and over, MSF clinics in Kamrangirchar, Dhaka, 2014-2023

| **Variable name** | **Variable type** | **Variable options** | **Description** |
| --- | --- | --- | --- |
| Date of consultation | Date | Not applicable | The date the patient had their consultation |
| Age | Numeric | Not applicable | Self-reported age of patient reported during their consultation |
| Sex | Categorical | Male, Female, Unknown/Unreported | Self-reported sex of patient reported during their consultation |
| Visit status | Categorical | New, Follow-up consultation | Type of consultation that the patient attended. A new consultation was defined as patients who came for the first time for a consultation. A follow-up consultation was defined as patients who came for subsequent appointments (scheduled or walk-ins) to reassess and manage the patient's ongoing occupational health condition. |
| Residence | Categorical | Lives inside the factory, Lives outside the factory | Self-reported patient residence either inside or outside of the factory |
| Type of factory | Categorical | Plastics, Metal, Garment, Leather, Tannery, Rubber, Embroidery, Chemical, Battery, Other | Self-reported factory in which the patient works |
| Primary diagnosis | Categorical | Musculoskeletal, Gastrointestinal, Dermatology, Respiratory, Injury, Ear, Nose, Throat, Dental Eyes and Head, Cardiovascular, Sexual and Reproduction health, Other chronic conditions, Other diagnoses | The primary diagnosis of the patient reported by the occupational health clinician |
| Work-related condition status | Categorical | Work-related, Non work-related | Outcome of the assessment by the occupational health clinician on whether the patient had a work-related or non work-related condition |
| Type of injury | Categorical | Cut/laceration, Crushing injury, Burn, Abrasion, Amputation, Broken bone, Bruise, Concussion, Other | Type of injury of the patient as reported by clinician |
| Mechanism of injury | Categorical | Struck, Fall, Burn, Caught in between, Other | Mechanism of injury reported by the patient |
| Nutritional status | Categorical | Malnourished, not malnourished | The nutrition status of the patient |
| Type of mental health disorder | Categorical | Mood-related problems, Behaviour-related symptoms, Family-related problems, Physical complaints, Neuropsychiatric-related symptoms, Social functioning, Other | The type of mental health disorder of the patient reported by the clinician |
| Precipitating event | Categorical | Domestic discord and family violence, Socioeconomic functioning, Disruption of family and relationships, Medical illness-related, Neuropsychiatric-related, Events related to abuse during detention, Events related to natural disasters, Deprivation, discrimination, and displacement, Sexual trauma or abuse, Others | The main event precipitating the presenting complaint |
